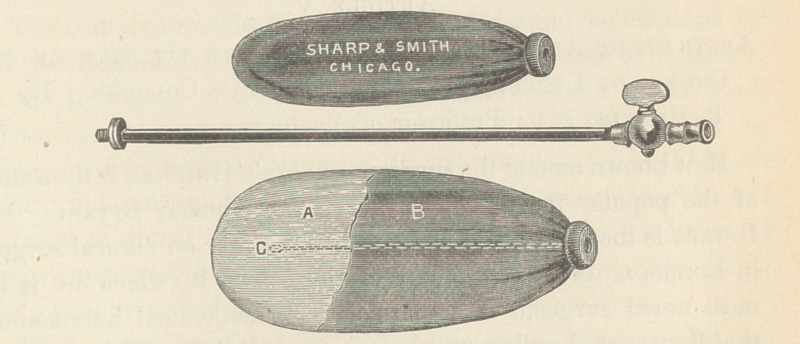# A New Uterine Dilator*This dilator was exhibited to the Chicago Society of Physicians and Surgeons, February 12th, 1877.

**Published:** 1879-11

**Authors:** J. O. Hobbs


### ARTICLE IV.

### A NEW UTERINE DILATOR.* J. O. HOBBS, M.D.

The idea of dilating the os uteri by means of an impervious sack or bag, introduced in a collapsed state and afterwards expanded by pumping in air or water, was known to Walbaum, who recommended the use of a pig's bladder for this purpose, about the middle of the last century. Barnes' dilators, which are simple rubber bags, are efficient in cases where the os will readily admit a large instrument and the resistance to be overcome is slight. Aside from being bulky and, therefore, difficult to introduce, they have another objectionable feature, viz., that of "pocketing," or distending indefinitely in the lines of least resistance when in use. With them the force used is expended in the dilatation of the rubber bag, the force exerted on the strictured part being measured by the elasticity of the walls of the sack.

Molesworth's dilators are rubber bags having threads imbedded longitudinally in their substance. They have the fault of pocketing in common with the Barnes' dilators, notwithstanding the claim of their inventor to the contrary. The longitudinal threads do not prevent their expansion laterally on either side of the constricted part, until the bursting point is reached.

Attempts have been made to obviate this difficulty by bandaging that part of the dilator which is external to the os, the portion of the sack within the uterus being left to expand indefinitely. It would be difficult to conceive of a more dangerous practice.

A case of rupture of the womb from the use of Molesworth's dilator has been reported. Molesworth's dilators are more powerful than Barnes' bags because they are made of heavier rubber, irrespective of the longitudinal threads. They require the use of a screw force pump to distend them.

The dilator which I have devised is essentially a sack of thin

---

* This dilator was exhibited to the Chicago Society of Physicians and Surgeons, February 12th, 1877.

inelastic fabric rendered impervious to air or water. As it is now constructed it consists of two sacks, one inclosing the other. The outer one is made of strong silk or linen, coated externally with rubber to prevent absorption. The inner sack is of the thinnest rubber. These are fastened to a perforated collar of metal from which a flexible probe is extended within the cavity. This collar makes a screw attachment to a small metallic tube provided with a stop-cock. This tube serves as a handle to the probe and can be connected at pleasure with a Davidson's or fountain syringe, or the air bulb of an atomizer.

When in use these dilators expand to a limited size of the os. The force applied is solely expended in overcoming the resistance. of the tissues constricting the sack. No power is lost in expanding the sack as is the case with the rubber bag, and for this reason they are very powerful dilators. With one of them I have dilated an artificial os of rubber of four times the thickness of one that could not be dilated with Molesworth's instrument. In making this experiment I used the common air bulb.

In practice I have used this dilator in two cases. The first instance was for the purpose of exploring the uterus of a woman who had suffered many years from metrorrhagia. The os fairly admitted the smallest sack which connected with a fountain syringe, suspended about five feet above the bed. At the end of three hours the os was dilated to the full size of the sack, permitting the removal of a fibroid polypus seven-eighths of an inch in diameter.

The patient was not anæsthetized during this gentle dilating process and complained but little of pain. The second case was one of abortion, in which the placenta and membranes had been retained for several days after expulsion of the fœtus. The same means to dilate the os were used as in the first case. Pressure was applied for a little more than an hour, shutting off the water now and then as the patient complained of regularly recurring pains which she described as being like those of the first stage of labor.

The advantages which I claim for these dilators are: 1st, Their small size and pliability when collapsed; 2d, Their fixed shape and size when distended; 3d, The direct application of force, which enables the operator to obtain satisfactory results with the least expenditure of power.

## Figures and Tables

**Figure f1:**